# Prospective evaluation of the usefulness of C-reactive protein in the diagnosis of neonatal sepsis in a sub-Saharan African region

**DOI:** 10.1186/2047-2994-1-22

**Published:** 2012-06-01

**Authors:** Boma A West, Oliemen Peterside, Rosemary O Ugwu, Augusta U Eneh

**Affiliations:** 1 Department of Paediatrics and Child Health, Braithwaite Memorial Specialist Hospital, Port Harcourt, Port Harcourt, Rivers State, Nigeria; 2 Department of Paediatrics and Child Health, Niger Delta University Teaching Hospital, Okolobiri, Bayels State, Nigeria; 3 Department of Paediatrics and Child Health, University of Port Harcourt Teaching Hospital, Alakahia, Rivers State, Nigeria

**Keywords:** Neonatal sepsis, C-reactive protein, Sub-Saharan Africa

## Abstract

**Background:**

Sepsis is one of the most common causes of morbidity and mortality in the newborn. Early diagnosis and treatment is vital to improve outcome. The present study was therefore carried out to determine the usefulness of C-reactive protein (CRP) for evaluation of neonatal sepsis in Port Harcourt, Nigeria in Sub-Saharan Africa.

**Method:**

Four hundred and twenty neonates with clinical suspicion of sepsis were prospectively studied over a 6 month period. Blood was obtained from each subject recruited for the qualitative estimation of CRP. Blood culture was used as gold standard for diagnosis of NNS.

**Results:**

Of 420 neonates studied, 196 (46.7%) had positive CRP while 181 (43.1%) had positive blood culture. The sensitivity, specificity, positive and negative predictive values of CRP were 74.0%, 74.1%, 68.4% and 79.0% respectively.

**Conclusion:**

The qualitative method of estimating CRP which is cheap and rapid has moderate sensitivity, specificity and negative predictive value.

## Introduction

Neonatal sepsis (NNS) and neonatal septicaemia are terms that have been used to describe the systemic response to infection and/or isolation of bacteria from the blood stream in the first 28 days of life [1]. Globally, sepsis accounts for 26% of all neonatal deaths [2] with 98% of these deaths occurring in developing countries [3]. Adequate and timely diagnosis of NNS remains an important challenge to the clinician. Blood culture is the gold standard for definitive diagnosis but it takes at least 48 hours by which time the infection may have progressed with important consequences on the morbidity and mortality of the neonate [4], especially if antibiotic treatment is not initiated immediately [5]. Initiation of antibiotic therapy before diagnostic results are available is recommended for neonates with clinical signs or risk factors of sepsis [6], but because the clinical signs of NNS are often non-specific, empiric antibiotic therapy may result in the treatment of as many as 30 uninfected neonates for every one who is eventually diagnosed to be infected [7-9]. Hence there is need for rapid screening test that can identify infected neonates at the time of initial assessment thus sparing the uninfected ones from unnecessary antibiotic therapy.

C-reactive protein is an acute phase protein which may be useful in the early diagnosis of NNS as it rises as much as a thousand fold within 4 to 6 hours of an inflammatory process [10,11]. As infection is the most likely cause of inflammation in the neonate, CRP has been shown to be useful in the diagnosis of neonatal sepsis. Upon resolution of the inflammation, CRP levels rapidly decline with an elimination half life of 19 hours [11,12]. Thus CRP level is also a useful marker in determining the duration of antibiotic therapy. Unlike blood culture, CRP level is not affected by prior antibiotic therapy [13], so may be particularly useful in Sub-Saharan Africa where a significant number of neonates may have been given antibiotics before presentation at the hospital.

CRP can be assayed quantitatively or qualitatively. The quantitative method is more widely used in developed countries [12]. It provides rapid, highly sensitive and specific results [14] but requires more time (about 15 to 30 minutes) and is more complex and expensive to perform [12]. The test kits may also not be readily available in some health centres in developing countries. The qualitative method provides rapid results within 15 minutes. However, it is less specific but has the advantage of being simple and easier to perform and interpret and as such can be performed at the patients bed side or side laboratory [14,15]. It is also less expensive and requires less skill. The qualitative method may therefore, be more feasible in resource poor centres, where poverty plays a prominent role in disease morbidity and mortality and where there may be no laboratory services or trained man-power for the investigation of neonates with suspected sepsis which is indeed the case in many areas of Sub-Saharan Africa.

To the knowledge of the authors, no study was found on the use of C-reactive protein in the detection of NNS in Nigeria. This study was therefore undertaken to ascertain the usefulness of C-reactive protein in the diagnosis and management of NNS.

## Materials and methods

Approval was obtained from the ethics and research committee of the University of Port Harcourt Teaching Hospital and informed consent was obtained from the parent or care givers of the neonates.

A prospective study was carried out in the Special Care Baby Unit (SCBU) of the University of Port Harcourt Teaching Hospital, Rivers State, Nigeria, within a 6 month period from May to November, 2007. All newborns with clinical suspicion or risk factors for sepsis were consecutively recruited into the study. Sepsis was suspected in the presence of clinical features like fever, respiratory distress, poor feeding, jaundice, hypothermia, convulsion, vomiting, irritability, lethargy and abdominal distension. Risk factors for sepsis included outborn delivery, perinatal asphyxia, preterm delivery, prolonged rupture of membranes, maternal peripartum pyrexia and foul smelling amniotic fluid.

Infants of mothers who had intrapartum antibiotics within 1 week of delivery as well as babies with prior antibiotic therapy for present illness before admission into the SCBU were excluded from the study.

C-reactive protein was estimated qualitatively using the Lorne CRP latex kit manufactured by the Lorne laboratories Limited (Great Britain) with catalogue number, 041100. The specific performance characteristics of the Lorne CRP latex reagent was standardized to detect serum CRP levels at or above 6 mg/l, which is considered the lowest concentration of clinical significance. Half a milliliter of venous blood was collected in plain bottles and centrifuged. C-reactive protein was estimated using a drop of undiluted serum placed onto the circle of the agglutination slide with the use of disposable pipettes provided in the kit. One drop of CRP latex reagent was added to the drop of serum and the broad end of the pipette was used to spread the latex reagent over the entire area of the test circle. The agglutination slide was gently tilted backwards and forwards approximately once every two seconds for two minutes. The results were read using the positive and negative controls as reference for agglutination. Visible agglutination of latex particles constituted a positive result which indicated a level of CRP > 6 mg/l while negative result was the reverse. After the results were read, the glass slide was rinsed with distilled water and air dried properly for re-use.

Blood culture was done for all recruited babies using two milliliter of venous blood collected from a peripheral vein after adequate skin preparation and before the commencement of antibiotics. The blood was aseptically introduced into aerobic and anaerobic culture media. The specimens were processed according to standard methods in the microbiology laboratory [16]. Inoculated blood culture media were considered negative if there was no growth after continuous incubation for up to 7 days, subcultures being made each day. Antibiotic sensitivity was done using Kirby-Bauer disc diffusion method [16].

The results of laboratory investigations and other relevant data such as age, sex, birth weight and gestational age as well as symptoms present and risk factors for sepsis of recruited babies were recorded in a proforma. The results were analysed using the statistical package, SPSS version 14.0. The sensitivity, specificity, positive and negative predictive values of CRP were calculated.

## Results

Of the 545 neonates admitted into the SCBU during the period of study, 420 (77.1%) were studied. There were 273 (65.0%) males and 147 (35.0%) females with mean gestational age (±SD) of 36.8 weeks (± 3.6) and mean birth weight (±SD) of 2.8 kg (±0.9). Three hundred and two (71.9%) of the study population had clinical features of sepsis, while 271 (64.5%) had risk factors for sepsis.

Of 420 neonates screened for sepsis, 181 (43.1%) had positive blood culture.

### Prevalence of positive C-reactive protein in neonates with suspected sepsis

Of the 420 neonates with suspected sepsis, 196 (46.7%) had positive CRP while 224 (53.3%) had negative CRP.

### Proportion of neonates with true positive and negative C-reactive protein using blood culture as gold standard

Of the 420 neonates studied, 181 (43.1%) had positive blood culture while 239 (56.9%) had negative blood culture. Of the 181 neonates with positive blood culture, 134 (74.0%) had positive CRP (True positive) while 47 (26.0%) had negative CRP (False negative). Of 239 neonates with negative blood culture, 177 (74.1%) had negative CRP (True negative) and 62 (25.9%) had positive CRP (False positive)

### Sensitivity, specificity, positive and negative predictive values of C-reactive protein

As shown in Table 1, the sensitivity, specificity, positive and negative predictive values were 74.0%, 74.1%, 68.4% and 79.0%, respectively.

**Table 1:** Sensitivity, Specificity, Positive and Negative Predictive Values of C-reactive protein using blood culture as gold standard

**Parameter**	**Formulae Used**	**Result (%)**
Sensitivity	134	74.0
	134 + 47	
Specificity	177	74.1
	177 + 62	
Positive predictive value	134	68.4
	134 + 62	
Negative predictive value	177	79.0
	177 + 47	

### Performance of CRP according to clinical features of sepsis

Table 2 shows the clinical features suggestive of sepsis. Of the 302 neonates with clinical features of sepsis, the most common was fever in 116 (38.4%) of them, followed by respiratory distress in 112 (37.1%). The CRP correctly identified all (100%) of the neonates with positive blood culture who presented with apnoea, vomiting and lethargy.

**Table 2:** Performance of CRP according to clinical features of sepsis

**Clinical features**	**Total** **n = 302**		**Positive blood culture within clinical feature**		**Positive CRP within positive blood culture**	
	No	%	No	%	No	%
Fever	116	38.4	52	44.8	42	80.8
Respiratory distress	112	37.1	48	42.9	37	77.1
Jaundice	81	26.8	27	33.3	22	81.5
Poor suck	75	24.8	39	52.0	29	74.4
Apnoea	27	8.9	7	25.9	7	100.0
Hypothermia	26	8.6	14	53.8	8	57.1
Convulsion	20	6.6	10	50.0	6	60.0
Vomiting	15	5.0	9	60.0	9	100.0
Irritability	10	3.3	6	60.0	5	83.3
Lethargy	10	3.3	6	60.0	6	100.0

### Performance of CRP according to predisposing factors for sepsis

As shown in Table 3, of the 271 neonates with predisposing factors for sepsis, the most common was out born delivery in 160 (59.0%) of them followed by perinatal asphyxia in 127 (46.9%). The CRP was able to correctly identify 83.3% and 81.8% of the neonates with positive blood culture who were born to mothers with foul smelling amniotic fluid and peri-partum pyrexia respectively.

**Table 3:** Performance of CRP according to predisposing factors for sepsis

**Predisposing factors**	**Total** **n = 271**		**Positive blood culture within predisposing factor**		**Positive CRP within positive blood culture**	
	No	%	No	%	No	%
Outborn Delivery	160	59.0	59	36.9	47	79.8
Perinatal Asphyxia	127	46.9	55	43.3	39	70.9
Prolonged Rupture of membranes	100	36.9	32	32.0	24	75.0
Prematurity	85	31.4	38	44.7	28	73.7
Foul smelling amniotic fluid	48	17.7	12	25.0	10	83.3
Peripartum pyrexia	22	8.1	11	50.0	9	81.8

### Performance of CRP in the neonates with proven sepsis

As shown in Table 4, of the 181 neonates with positive blood culture, the most common organism isolated was Klebsiella pneumonia in 105 (58.0%), followed by staphylococcus aureus in 37 (20.4%). The CRP was able to correctly identify all cases (100.0%) of neonatal sepsis caused by Escherichia coli, Proteus spp, Enterococcus spp, Staphylococcus epidermidis and Streptococcus spp.

**Table 4:** Performance of CRP in the neonates with proven sepsis

**Organisms isolated**	**Total** **n = 181**		**Positive CRP within positive blood culture**	
	No	%	No	%
*Klebsiella pneumonia*	105	58.0	100	95.2
*Staphylococcus aureus*	37	20.4	35	94.6
*Escherichia coli*	14	7.7	14	100.0
*Proteus spp*	9	5.0	9	100.0
*Pseudomonas aerugenosa*	8	4.4	5	62.5
*Enterococcus spp*	3	1.7	3	100.0
*Staphylococcus epidermidis*	3	1.7	3	100.0
*Streptococcus spp*	2	1.1	2	100.0

## Discussion

The prevalence of blood culture proven sepsis in the present study was 43.1%. This is similar to the 41.7% reported by Chako and Sohi [17], 42% by Mustafa et al. [15] and 47.5% by Roy et al. [18]. It is however higher than the 36.4% reported by Antia-Obong and Utsalo [19], 28% by Zeeshan et al. [20], 14% by Manucha et al. [21] and 10.7% by Ugochukwu [22].

The commonest organism isolated was klebsiella pneumonia followed by staphylococcus areus. From 1974 to 1978, Omene [3] in Benin city found Escherichia coli to be the predominant bacterial isolate in neonatal sepsis. Antia Obong and Utsalo [19] in Calabar reported Staphylococcus aureus as the predominant bacterial isolate from 1985 to 1987. Ugochukwu [23] also found Staphylococcus aureus as the predominant bacterial isolate in neonatal sepsis in Nnewi Nigeria, from 1998 to 2001. This shows a changing pattern of bacterial isolates over the years in the Southern region of Nigeria. Similar to the finding in the present study, Roy et al. [18] in India reported Klebsiella spp as the predominant bacterial isolate in neonatal sepsis.

Among the clinical features of neonatal sepsis in the present study, CRP performance was highest in neonates with apnoea, vomiting and lethargy and lowest in those with hypothermia and convulsion. The lower levels in neonates with hypothermia and convulsion may be due to the fact that there are other commoner causes of neonatal hypothermia and convulsions other than sepsis.

Among the risk factors for neonatal sepsis, CRP performance was highest in neonates born to mothers with foul smelling amniotic fluid, followed by peri-partum pyrexia. This is similar to findings by Mathai et al. [23] in Tamil, Nadu who reported a significant association between maternal peri-partum pyrexia and neonatal positive CRP levels. Unlike the present study however, they found no significant association between foul smelling amniotic fluid and positive CRP levels.

In the present study, CRP identified 134 out of 181 neonates who had culture-proven sepsis, with sensitivity of 74.0% and a negative predictive value of 79.0%, implying that close to three quarters of neonates with suspected sepsis will be correctly diagnosed using CRP. This means that one out of every four neonates with sepsis will be missed. This is much too high to base the decision not to start empirical antibiotics for a neonate with suspected sepsis. Particularly as CRP was only tested to predict positive blood culture which may represent only a proportion of neonates with sepsis [24], especially if the patient had been on antibiotic therapy before presentation as is common in Sub-Saharan Africa. A negative CRP, however can be useful in aiding the decision to discontinue antibiotics especially if the neonate has no clinical feature of sepsis. Kashabi et al. [13] demonstrated that CRP can be a useful guide in making a decision to discontinue antibiotic therapy, thus facilitating early discharge with significantly reduced cost, complications of treatment and family anxiety.

Several authors in different settings have reported different performances for usefulness of CRP in the diagnosis and management of neonatal sepsis. The main differences and the CRP performances are as outlined in Table 5. Nuntnarumit et al. [25] in Bangkok Thailand, reported the highest sensitivity, specificity, positive predictive values and negative predictive value. This is probably due to the quantitative sampling method which they used as compared to the qualitative method used in the present study. Also as shown in Table 5, authors who used the quantitative method for measurement of CRP reported higher performance values than those who used the qualitative method.

**Table 5:** Performance of CRP in different settings

**Area**	**Method**	**Sample size**	**Performance**			
			**Sensitivity**	**specificity**	**PPV**	**NPV**
Bangkok (Thailand) [25]	Quantitative	76	100.0%	94.0%	91.6%	100.0%
Brazil [26]	Quantitative	69	78.6%	87.5%	91.7%	70.0%
Tamil, Nadu (India) [23]	Semi-quantitative	250	80.0%	60.0%	7.7%	98.6%
Rawalpindi (Pakistan) [20]	Qualitative	100	85.6%	95.0%	82.7%	95.9%
Delhi (India) [21]	Qualitative	150	76.0%	79.0%	37.0%	96.0%
*Port Harcourt (Nigeria)	**Qualitative**	**420**	**74.0%**	**74.1%**	**68.4%**	**79.0%**

*present study.

## Conclusion

The qualitative method of CRP estimation, which is a rapid, inexpensive and simple test to perform, was found to have moderate sensitivity, specificity and NPV of 74.0%, 74.1% and 79.0%, respectively. This implies that CRP would correctly identify close to three quarters of neonates with sepsis and would have 79.0% probability in excluding sepsis.

The C-reactive protein may therefore, help in the early detection of neonatal sepsis while awaiting blood culture results. CRP may also be invaluable in the management of neonatal sepsis in resource poor centres where facilities for blood culture may not be readily available.

## Competing interests

The authors declared that they have no competing interest.

## Authors contributions

BAW conceived of the idea for the study, designed the proforma and wrote the initial manuscript. OP assisted in recruitment of patients, analysis of data and writing of the manuscript. ROU and AUE both revised the manuscript and made significant intellectual contributions. All authors read and approved the final manuscript.
